# Population Pharmacokinetic Analysis and Simulation of Alternative Dosing Regimens for Biosimilars to Adalimumab and Etanercept in Patients with Rheumatoid Arthritis

**DOI:** 10.3390/pharmaceutics16060702

**Published:** 2024-05-23

**Authors:** Stephanie F. Ling, Kayode Ogungbenro, Adam S. Darwich, Amirah Binti Mohammad Ariff, Nisha Nair, James Bluett, Ann W. Morgan, John D. Isaacs, Anthony G. Wilson, Kimme L. Hyrich, Anne Barton, Darren Plant

**Affiliations:** 1 Centre for Genetics and Genomics Versus Arthritis, Centre for Musculoskeletal Research, The University of Manchester, Manchester M13 9PT, UK; 2 NIHR Manchester Biomedical Research Centre, Manchester University NHS Foundation Trust, Manchester Academic Health Science Centre, Manchester M13 9WL, UK; 3 Centre for Applied Pharmacokinetic Research, The University of Manchester, Manchester M13 9PT, UK; 4 Division of Health Informatics and Logistics, KTH Royal Institute of Technology, 11428 Stockholm, Sweden; 5 School of Medicine, University of Leeds, Leeds LS2 9JT, UK; 6 NIHR Leeds Biomedical Research Centre, Leeds Teaching Hospitals NHS Trust, Leeds LS7 4SA, UK; 7 NIHR In Vitro Diagnostic Co-Operative, Leeds Teaching Hospitals NHS Trust, Leeds LS9 7TF, UK; 8 Translational and Clinical Research Institute, Newcastle University, Newcastle-upon-Tyne NE1 7RU, UK; 9 Musculoskeletal Unit, Newcastle-upon-Tyne Hospitals NHS Foundation Trust, Newcastle-upon-Tyne NE7 7DN, UK; 10 School of Medicine and Medical Science, Conway Institute, University College Dublin, Dublin 4 Belfield, Ireland; 11 Centre for Epidemiology Versus Arthritis, Centre for Musculoskeletal Research, The University of Manchester, Manchester M13 9PT, UK

**Keywords:** rheumatoid arthritis, pharmacokinetics, population pharmacokinetics, simulation, biologics, biosimilars

## Abstract

Efficacy to biologics in rheumatoid arthritis (RA) patients is variable and is likely influenced by each patient’s circulating drug levels. Using modelling and simulation, the aim of this study was to investigate whether adalimumab and etanercept biosimilar dosing intervals can be altered to achieve therapeutic drug levels at a faster/similar time compared to the recommended interval. RA patients starting subcutaneous Amgevita or Benepali (adalimumab and etanercept biosimilars, respectively) were recruited and underwent sparse serum sampling for drug concentrations. Drug levels were measured using commercially available kits. Pharmacokinetic data were analysed using a population approach (popPK) and potential covariates were investigated in models. Models were compared using goodness-of-fit criteria. Final models were selected and used to simulate alternative dosing intervals. Ten RA patients starting the adalimumab biosimilar and six patients starting the etanercept biosimilar were recruited. One-compartment PK models were used to describe the popPK models for both drugs; no significant covariates were found. Typical individual parameter estimates were used to simulate altered dosing intervals for both drugs. A simulation of dosing the etanercept biosimilar at a lower rate of every 10 days reached steady-state concentrations earlier than the usual dosing rate of every 7 days. Simulations of altered dosing intervals could form the basis for future personalised dosing studies, potentially saving costs whilst increasing efficacy.

## 1. Introduction

Rheumatoid arthritis (RA) is a chronic, autoimmune arthritis with multisystem involvement; permanent joint damage can occur if inflammation is not controlled promptly [1]. Biologic disease-modifying anti-rheumatic drugs (bDMARDs) are commonly used in patients with moderately-to-highly active RA; their prescription in the UK has increased since a reduction in thresholds to commence tumour necrosis factor inhibitors (TNFi, a class of bDMARD) [2]. The TNFi agents adalimumab and etanercept are self-administered by patients in pre-filled auto-injectors at set dosing intervals. Since 2013, a number of biosimilar bDMARDs have also been licensed for prescription for RA [3].

An inefficacy rate of ≤40% exists in TNFi [4]; one cause of inefficacy is sub-therapeutic drug levels [5]. Research has consistently demonstrated a significant association between adalimumab/etanercept drug levels and treatment response [5]. There is substantial variability in circulating drug levels between patients [5], referred to as between-subject variability (BSV) in drug exposure [6]. Pharmacokinetic (PK) models aim to describe how drug concentrations vary over time. In basic compartmental analysis, many PK models are structurally defined using “compartments”, with each representing a hypothetical part of the body. A single compartment model represents a well-mixed and kinetically homogeneous body compartment, enabling the drug to be described with a single representative concentration at any given time point [7].

Individual PK studies employ detailed drug concentration−time data, which are typically analysed using non-compartmental models. By contrast, population (pop) PK modelling incorporates concentration−time data from multiple individuals and does not require rich or balanced drug concentration sampling [8]. PopPK modelling represents an approach for analysing PK study data; it can be used to analyse sparse data where the standard approach of non-compartmental analysis cannot be used. Typically, popPK models are used to quantify variability (e.g., BSV) in a population study and link this to sources (covariates) that can influence PK (e.g., age, sex). A number of popPK studies have been carried out in patients with RA starting the TNFi adalimumab [9,10] and etanercept [11,12,13,14]. However, only one popPK study has been published using an etanercept biosimilar in healthy subjects only [15], and no adalimumab biosimilar studies have been published.

This study aimed to carry out PK studies in RA patients starting biosimilars to adalimumab and etanercept (Amgevita and Benepali, respectively) in a cohort of real-world patients, analysing the data using a popPK modelling approach. Parameters from the models were then used to simulate PK profiles using altered dosing intervals to achieve maximal effect and/or steady-state at accelerated time points.

## 2. Materials and Methods

### 2.1. Study Aims and Design

1. To carry out PK studies in real-world patients with RA starting Amgevita and Benepali (adalimumab and etanercept biosimilars, respectively) using a popPK modelling approach.
2. To use parameters from the above popPK models to simulate PK profiles using altered dosing intervals to determine whether maximal effect and/or steady-state plasma drug levels can be attained at accelerated time points.

### 2.2. Study Setting

Patients with RA were recruited from rheumatology clinics in three centres based in Greater Manchester, UK, into the Biologics in RA Genetics and Genomics Study Syndicate [16] (BRAGGSS, Research Ethics Committee reference: 04/Q1403/37), a prospective multi-centre observational study based in the UK.

### 2.3. Study Participants

Patients with RA, according to the 1987 American College of Rheumatology classification criteria, were recruited to the Personalised Dosing sub-study of BRAGGSS (BRAGGSS-PD) in compliance with the Declaration of Helsinki. All participants gave written informed consent. As well as a clinical diagnosis of RA, inclusion criteria required participants to be starting Amgevita or Benepali (adalimumab and etanercept biosimilars, respectively), be bDMARD-naïve, have a pre-treatment Disease Activity Score of 28 Joints (DAS28) ≥ 5.1 (indicating high disease activity) at the time of recruitment and be aged ≥ 18 years. At the time of the study, this DAS28 threshold was required by the National Institute for Health and Clinical Excellence (NICE) in England and Wales for patients to qualify to start a bDMARD. Patients were excluded if they did not fulfil all inclusion criteria. Participants were recruited between January 2019 and August 2021 and were followed up for a total of 12 weeks. The adalimumab and etanercept biosimilars were self-administered subcutaneously by patients at licensed doses of 40 mg every 14 days and 50 mg every 7 days, respectively. These specific biosimilars were selected for study because they are the two agents that have been approved locally for prescription by National Health Service (NHS) rheumatology departments in Greater Manchester, UK, where this study was carried out.

### 2.4. Sampling Schedules

Optimal sampling time intervals were proposed using previously defined popPK models from the literature in PopDes software v1, an application software that can be utilised for determining optimal sampling times or windows for popPK studies [17]; further information on this software is detailed in the Supplementary Methods. All samples were collected by the same investigator, and all doses given at study visits were also witnessed by that same investigator.

For the adalimumab biosimilar, a previous PK model derived for adalimumab originator [10] was used to simulate multiple dosing interval models. The optimal sampling design was determined to be at baseline (pre-treatment), 1 hour post-first dose, then 2, 4, 6 and 12 weeks post-first dose. Samples subsequent to the second sample were taken directly before the next dose in order to ensure true trough level sampling. For the etanercept biosimilar, previous PK models derived for etanercept originator [12,13,18] were utilised for simulation. The optimal sampling design was determined to be as for the adalimumab biosimilar, with an additional sampling time point at 6 days post-first dose.

### 2.5. Sample Processing

All participant samples were processed and stored at the Centre for Musculoskeletal Research (CfMR), the University of Manchester, Manchester, UK, and were processed by CfMR laboratory staff. All sample blood tubes were spun at 1720× *g* for 10 min, then serum was extracted into aliquots and stored in −150 °C freezers.

### 2.6. Measurement of Drug Levels

Drug level measurements were carried out by CfMR laboratory staff using commercially available ELISA-based test kits produced by Grifols International, SA (Barcelona, Spain). The Promonitor^®^-ADL-1DV kit was used to measure the adalimumab biosimilar drug levels and the Promonitor^®^-ETN-1DV kit was used to measure the etanercept biosimilar drug levels. Both had previously been validated [19,20]. Standard laboratory equipment and a spectrophotometer (SpectraMax^®^ Plus 384 Microplate Reader, Molecular Devices, LLC, San Jose, CA, USA) were used during the experimental procedure. Samples were defrosted for two hours at room temperature prior to thorough mixing before the experimental procedure.

Serum drug levels were measured in triplicate using 96-microwell ELISA plates, which were pre-coated with anti-adalimumab and anti-etanercept human monoclonal antibody according to which drug was being measured. Patient samples were diluted to 1:50 concentration using a dilution buffer and were transferred to separate wells. Pre-diluted calibration samples and positive and negative controls were also included for the purposes of quantification of results and quality control; these were also transferred to separate wells. Any drug present in the patient samples, calibration samples and controls became bound to the immobilised anti-drug antibodies during an incubation period of one hour at room temperature. Following incubation, any unbound material was removed by washing the wells with a 20× buffer containing phosphate-buffered saline and tween-20. Each well was then loaded with a second horseradish peroxidase-labelled anti-drug monoclonal antibody to form a sandwich complex. The plate was incubated for a further one hour at room temperature to allow the labelled antibody to bind to the drug attached to the microwells. Unbound enzyme-labelled antibody was again washed away with wash buffer, and a substrate of pre-diluted stabilised tetramethylbenzidine was added to measure enzyme activity. After 15 min, a stop reagent of pre-diluted sulphuric acid solution was added to halt the reaction. Colour intensity as a result of the enzymatic reaction was measured in triplicate using a spectrophotometer at wavelength 450 nm. The generated optical density values were proportional to the drug concentration in each sample.

Softmax Pro 7 software (compatible with the SpectraMax^®^ Plus 384 Microplate Reader, Molecular Devices, LLC, San Jose, CA, USA) was used to interpolate the optical density values and determine drug level concentrations. Interpolated values were multiplied by the dilution factor (×50) to obtain drug levels in patient samples.

### 2.7. PopPK Analysis

(i) Software

PK data were analysed using a population approach with Monolix v.2019R2 software (Lixoft, Antony, France). This is a non-linear mixed-effects modelling software package based on the stochastic approximation expectation maximisation (SAEM) algorithm. Monolix works by optimising the maximum likelihood to produce optimal population parameter values; final estimates maximise the likelihood of data, given the model. The default simulated annealing option for SAEM was used in order to maintain a larger parameter space. A maximum of 500 iterations was set to ensure the best possible convergence. For medication doses that were self-administered by patients outside of study visits, nominal dose timings were used for the purpose of modelling.

(ii) PopPK analysis

For each drug studied, one-, two- or three-compartment mammillary models assuming first-order absorption and elimination were tested. Estimated PK parameters were given as apparent values due to extravascular administration via the subcutaneous route. PK parameters were parameterised as clearance (CL) and volume of distribution (V_D_). Structural models were compared using the Akaike information criterion (AIC). BSV in PK parameters was described using an exponential model. For parameters where BSV could not be estimated, this was removed from the analysis and, therefore, only typical individual values were estimated. Correlations between parameters were tested. Additive, proportional or combined additive and proportional models were tested for residual unexplained variability (RUV). The following four covariates were investigated: age and body weight (continuous covariates) and sex and concurrent conventional synthetic disease-modifying anti-rheumatic drug (csDMARD) therapy (binary covariates). Covariate models were compared using both −2 log-likelihood (−2LL) and AIC. Models with the lowest significant −2LL value (assessed using a likelihood ratio χ^2^ test, LRT) and the lowest AIC, with the simplest combination of covariates and between-variable correlations, were selected.

Goodness-of-fit (GOF) was visually assessed using plots of:

- Population-predicted (PRED) and individual-predicted (IPRED) measurements versus observed measurements (DV).
- IPRED and DV versus time.
- Residuals, represented in plots of:
  ⚬ Population weighted residual distributions (PWRES).
  ⚬ Individual weighted residual distributions (IWRES).
  ⚬ Normalised prediction distribution errors (NPDE). Distribution was tested using the Shapiro–Wilk test at a level of α = 0.05.

(iii) Simulation of altered dosing intervals

Using model parameters estimated from final popPK models for each drug, simulations of altered dosing intervals were carried out in the R v.4.0.5 [21] base package. The ggplot2 [22] package was used to visualise simulations. Simulations of altered dosing intervals (with the same dose of pre-filled syringe as licensed) were carried out. For the adalimumab biosimilar, dosing intervals of 40 mg every 7 days, 14 days (usual interval) and 21 days were simulated. For the etanercept biosimilar, intervals of 50 mg every 5 days, 7 days (usual interval) and 10 days were simulated.

All methods above are described in more detail elsewhere [23].

## 3. Results

### 3.1. Cohort Characteristics

Ten RA patients commencing Amgevita (adalimumab biosimilar) were recruited (Table 1); nine were female and one was male. All patients were white. The median age was 50.5 years (interquartile range, IQR, 46-61) and the median pre-treatment DAS28 was 5.71 (IQR 5.20–6.09). Six RA patients commencing Benepali (etanercept biosimilar) were recruited (Table 2); four were female and two were male. One patient was West African and the remaining patients were white. The median age was 57.5 years (IQR 56–59) and the median pre-treatment DAS28 was 5.33 (IQR 4.96–5.58). Participant numbers were below those planned, as recruitment was curtailed due to the COVID-19 pandemic.

### 3.2. PopPK Modelling

A total of 58 serum samples of the adalimumab biosimilar and 40 serum samples of the etanercept biosimilar drug concentrations were available for analysis. Drug concentration values at each time point are available in Supplementary Tables S1 and S2. One-compartment PK models were found to be sufficient to describe the data in popPK modelling of participants initiating both the adalimumab and etanercept biosimilars. Combined additive and proportional models were used to describe the residual errors in the data.

In the adalimumab biosimilar model, PK parameters estimated are presented in Table 3. No covariates were included, as covariates tested only demonstrated a modest improvement in -2LL and AIC whilst complicating the sparse-sampling model with redundant variables. Due to a large percentage relative standard error (RSE%) from estimation of ka (minimum 622%), this value was fixed to 0.01167 hour^-1^, as per Ternant et al. [10], and the random effect (BSV) was not estimated for this parameter. Plots were generated of predicted versus observed measurements for the adalimumab biosimilar serum concentrations, demonstrating that PK parameters described the data adequately (Supplementary Figure S1). Diagnostic plots are presented in Supplementary Figures S2 and S3. 

In the etanercept biosimilar model, the PK parameters estimated are presented in Table 4. No covariates were included due to the same reasons as for the adalimumab biosimilar model. Due to a large RSE% from estimation of ka (minimum 1.070%), this value was fixed to 0.0396 hour-1, as per Korth-Bradley et al. [24], and the BSV was not estimated for this parameter. Additionally, the model had a large RSE% for estimated V_D_, so the BSV estimation was removed from this parameter. Finally, the additive error standard deviation (SD) was fixed to 0.0001 to ensure model stability. Plots were generated of predicted versus observed measurements for the etanercept biosimilar serum concentrations, demonstrating that PK parameters described the data adequately (Supplementary Figure S4). Other diagnostic plots are presented in Supplementary Figures S5 and S6.

### 3.3. Simulation of Altered Dosing Intervals

**Figure 1:**
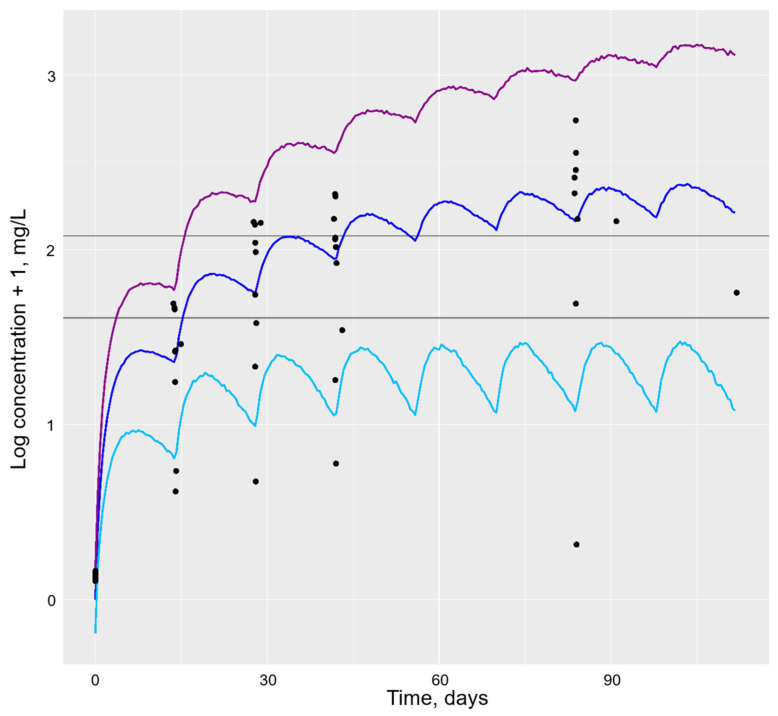
Simulation of the adalimumab biosimilar PK profile for 10,000 individuals using the final popPK model. Legend: Horizontal grey lines represent the adalimumab therapeutic window of 5–8 mg/L proposed by Pouw et al. [25]. The dark blue line represents the population median, the light blue line represents the 5th percentile and the purple line represents the 95th percentile of the population. The black dots represent actual population drug concentration values.

**Figure 2:**
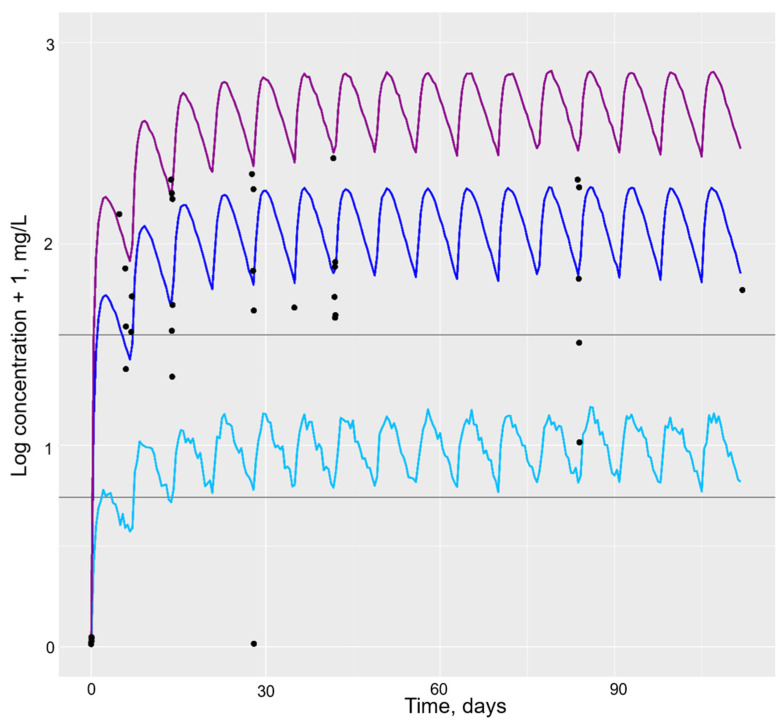
Simulation of the etanercept biosimilar PK profile for 10,000 individuals using the final popPK model. Legend: Horizontal grey lines represent the etanercept therapeutic window of 2.1–4.7 mg/L proposed by Jamnitski et al. [26]. The dark blue line represents the population median, the light blue line represents the 5th percentile and the purple line represents the 95th percentile of the population. The black dots represent actual population drug concentration values.

Initially, simulations of 10,000 individuals were carried out using parameter estimates and the final popPK models for the adalimumab and etanercept biosimilars. Typical individual profiles, as well as the median, 5th and 95th percentiles, were simulated using the usual dosing intervals of the adalimumab biosimilar 40 mg every 14 days (Figure 1) or the etanercept biosimilar 50 mg every 7 days (Figure 2), respectively. Simulated profiles were overlaid with observed plasma concentration data; these demonstrated visually that simulated values agreed well with the data.

**Figure 3:**
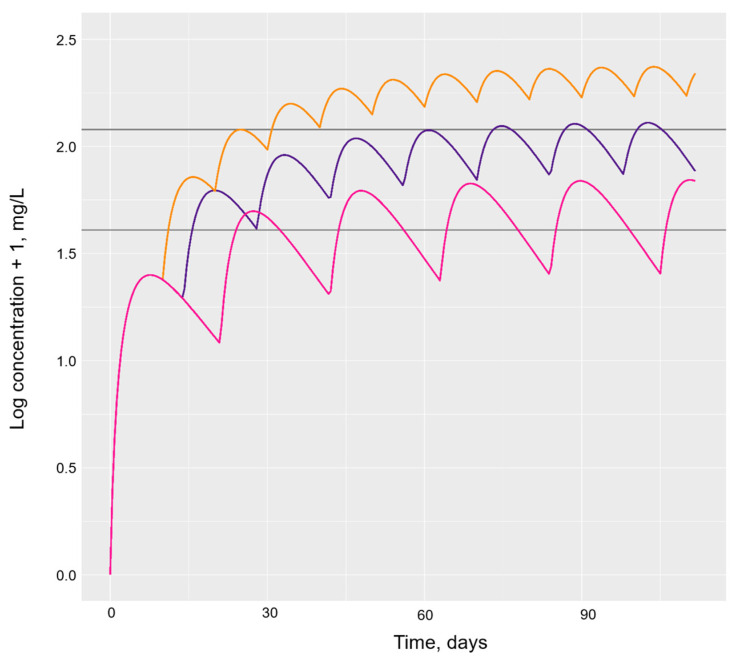
Simulated usual and alternative dosing intervals of the adalimumab biosimilar for a typical patient. Legend: Horizontal grey lines represent the adalimumab therapeutic window of 5–8 mg/L proposed by Pouw et al. [25]. The purple line represents the usual adalimumab biosimilar dosing interval of 40 mg every 14 days, the orange line represents a dosing interval of 40 mg every 7 days and the pink line represents 40 mg every 21 days.

Once this had been established, alternative dosing regimens were additionally simulated for typical individuals on the adalimumab biosimilar (Figure 3). Both the usual dose rates of 40 mg every 14 days and the increased dose rate of 40 mg every 7 days achieved steady-state drug concentrations within the therapeutic window of adalimumab, defined as between 5–8 mg/L [25]. However, the reduced dose rate of 40 mg every 21 days from initiation did not achieve steady-state concentrations within this proposed window. Time to steady-state drug concentrations had negligible differences between the administration of adalimumab biosimilar 40 mg every 7 or 14 days.

**Figure 4:**
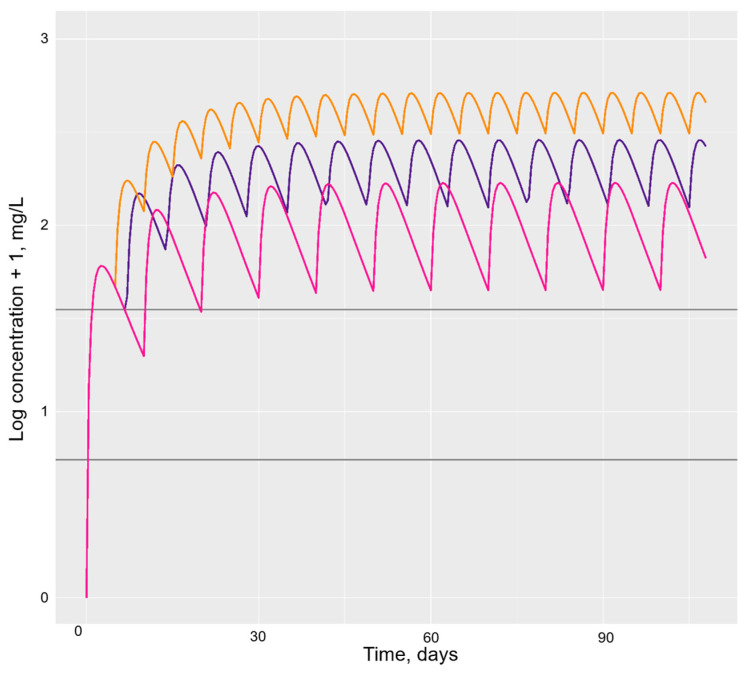
Simulated usual and alternate dosing intervals of the etanercept biosimilar for a typical patient. Legend: Horizontal grey lines represent the etanercept therapeutic window of 2.1–4.7 mg/L proposed by Jamnitski et al. [26]. The purple line represents the usual etanercept biosimilar dosing interval of 50 mg every 7 days, the orange line represents a dosing interval of 50 mg every 5 days and the pink line represents 50 mg every 10 days.

Alternative dosing regimens were also simulated for typical individuals on the etanercept biosimilar (Figure 4). All simulated doses achieved steady-state drug concentrations well above the therapeutic window of etanercept, defined as between 2.1–4.7 mg/L [26]. Time to steady-state drug concentrations had negligible differences between the three simulated dosing regimens of the etanercept biosimilar, although this was marginally quicker with an interval of every 10 days on visual inspection.

## 4. Discussion

Using 58 drug concentration samples from ten patients starting the adalimumab biosimilar Amgevita and 40 samples from six patients starting the etanercept biosimilar Benepali, collected over a 12-week period for each patient, popPK models were developed for the study population. Parameter estimates were similar to those from previous studies [10,24], and, alongside acceptable visual checks, this meant that the model fit was determined to be satisfactory. Therefore, values were used to successfully simulate models illustrating altered dosing intervals of both drugs compared to usual dosing in a typical individual. A simulation of dosing Benepali at a lower rate of every 10 days showed drug concentrations reaching steady-state quicker than the usual dosing rate of every 7 days (Figure 4), which has potential cost-saving implications.

There is only one other popPK study of subcutaneously administered adalimumab in RA patients, conducted by Ternant et al. [10], and this study was the basis for the corroboration of estimated PK parameters. In contrast to the present study, Ternant’s was a post hoc analysis of a single-centre observational study carried out over 52 weeks, and it used adalimumab originator compound. No popPK analyses have been published for the adalimumab biosimilar Amgevita prior to this study.

The current study is the first published popPK analysis of patients with RA starting the etanercept biosimilar Benepali, and, furthermore, it is the first using the usual dosing regimen of 50 mg subcutaneously every 7 days; other studies have used mixed dosing regimens with a mixture of intravenous and subcutaneous administration [11,12,13]. The findings of the current study broadly agree with those of Korth-Bradley et al., who carried out popPK analysis in a cohort of healthy controls who received only a single dose of etanercept 25 mg subcutaneously (non-standard dose) [24]. However, whilst subcutaneous delivery of monoclonal antibodies, such as etanercept, is now being used widely, the variations in bioavailability between intravenous and subcutaneous routes and the mechanisms of these variations remain poorly understood [27]. The finding that dosing intervals could potentially be reduced to once every 10 days is interesting; however, given the small sample size, this requires validation in a larger study. Furthermore, because of the size of the study, BSV may not have been described adequately.

Interestingly, patients on the adalimumab biosimilar had an increased median body weight compared with those on the etanercept biosimilar (85.5 kg versus 70.5 kg, respectively). Simulation results for patients receiving the two different biosimilars could, therefore, have been affected by the characteristics of the populations studied, as the adalimumab biosimilar population may have experienced impaired subcutaneous drug absorption secondary to increased adiposity. Furthermore, previous studies have demonstrated that TNFi treatment response is reduced with increased BMI [28].

The majority of patients in the UK are commenced on a biosimilar version of TNFi; therefore, it is important to explore the PK profile of these drugs in patients with RA and how this may influence disease outcome. This study was designed specifically for this purpose and was not carried out as part of a post hoc analysis. The patients were real-world patients managed in UK NHS rheumatology departments, rather than clinical trial participants, so the drug concentrations obtained are likely to be more reflective of day-to-day clinical practice. All samples were collected by the same investigator and delivered to the central processing laboratory within 24 h of blood draw. All blood draws and witnessed dose administrations were documented to the nearest minute by that same investigator, maximising the accuracy of the popPK models. Witnessing of doses at each study visit ensured true trough drug concentrations. Furthermore, because sampling and dose witnessing were all carried out by a single investigator, this ensured no deviations from protocol and consistency of time reporting.

One limitation of this study is that samples were not tested for anti-drug antibodies. Whilst anti-etanercept antibodies occur infrequently, anti-adalimumab antibodies are associated with decreased drug concentrations and reduced rates of treatment response [5]. Due to the low sample numbers, which were ultimately limited due to multiple UK lockdowns in response to the COVID-19 pandemic, recruitment was below the optimum total of participants. Therefore, including anti-drug antibody seropositivity as a covariate, as with the other covariates tested in this study, would have been unlikely to improve model fit, and hence, was not tested.

Due to low participant numbers, ka had to be fixed in both the adalimumab and etanercept biosimilar models and covariates were not identified, so popPK models may not be completely representative of the study populations. Furthermore, covariate selection can be problematic based on low participant numbers, i.e., although certain covariates can appear as significant/non-significant in a given model, these findings remain uncertain due to sample size [29]. For example, due to the skew between female and male participants, we cannot distinguish between BSV and the effect of sex. Previous work has suggested that a sample size of 20–30 participants per drug would provide a more accurate parameter estimation using a popPK approach [30]. The low study numbers are not necessarily a negative though, as this study itself adds valuable information and data in a space where both are very limited. To this end, it is a rational approach to establish a pilot study before carrying out any larger studies.

## 5. Conclusions

In conclusion, popPK models for the TNFi biosimilars Amgevita and Benepali were successfully developed in populations of real-world RA patients. Findings from simulations of altered dosing intervals could form the basis for future personalised dosing studies for these two agents.

## Figures and Tables

**Table 1:** Patient characteristics at baseline, prior to treatment with the adalimumab biosimilar (*n* = 10).

Characteristic	Statistic
Female sex, *n* (%)	9 (90.00)
Age (years), median [IQR]	50.5 [46–61]
Body weight (kg), median [IQR]	85.5 [66–111]
Body mass index (kg/m^2^), median [IQR]	36.2 [28.7–43.4] (2 missing observations)
Concurrent csDMARD, *n* (%)	8 (80.00)
DAS28, median [IQR]	5.71 [5.20–6.09]

Abbreviations: Conventional synthetic disease-modifying anti-rheumatic drug (csDMARD), disease activity score of 28 joint counts (DAS28), interquartile range (IQR).

**Table 2:** Patient characteristics at baseline, prior to treatment with the etanercept biosimilar (*n* = 6).

Characteristic	Statistic
Female sex, *n* (%)	4 (66.67)
Age (years), median [IQR]	57.5 [56–59]
Body weight (kg), median [IQR]	70.5 [69–84]
Body mass index (kg/m^2^), median [IQR]	24.6 [22.8–28.2] (1 missing observation)
Concurrent csDMARD, *n* (%)	4 (100.00) (2 missing observations)
DAS28, median [IQR]	5.33 [4.96–5.58]

Abbreviations: Conventional synthetic disease-modifying anti-rheumatic drug (csDMARD), disease activity score of 28 joint counts (DAS28), interquartile range (IQR).

**Table 3:** Population pharmacokinetic (popPK) model parameter estimates for the adalimumab biosimilar.

Parameter (Units)	Definition	Estimate	Relative Standard Error (RSE, %)
V_D_ (L)	Apparent volume of distribution	9.19	12.7
CL (L/h)	Apparent clearance	0.00283	23.3
ka (/h)	Rate constant for absorption	0.1167	Fixed
ωV_D_ (%)	Coefficient of variation (CV) of between-subject variability (BSV) on V_D_	15.60	141.0
ωCL (%)	CV of BSV on CL	68.90	24.8
σprop (%)	Standard deviation (SD) of proportional residual error	26.00	15.7
σadd (mg/L)	Standard deviation of additive residual error	10.80	16.0

The relative standard error (RSE, %) was calculated as: RSE = (estimate/standard error) × 100. Abbreviations: Additive error (add), between-subject variability (BSV), clearance (CL), coefficient of variation (CV), proportional error (prop), proportional rate constant for absorption (ka), standard deviation (SD), volume of distribution (V_D_).

**Table 4:** Population pharmacokinetic (popPK) model parameter estimates for the etanercept biosimilar.

Parameter (Units)	Definition	Estimate	Relative Standard Error (RSE, %)
V_D_ (L)	Apparent volume of distribution	7.76	18.2
CL (L/h)	Apparent clearance	0.0404	10.7
ka (/h)	Rate constant for absorption	0.0396	Fixed
ωCL (%)	Coefficient of variation (CV) of between-subject variability (BSV) on CL	0.173	61.0
σprop (%)	CV of proportional residual error	0.46	13.6

The relative standard error (RSE, %) was calculated as: RSE = (estimate/standard error) × 100. Abbreviations: Between-subject variability (BSV), clearance (CL), coefficient of variation (CV), proportional error (prop), rate constant for absorption (ka), volume of distribution (V_D_).

## Data Availability

The data presented in this study are available on request from the corresponding author due to patient confidentiality.

## Supplementary Materials

The following supporting information can be downloaded at: https://www.mdpi.com/article/10.3390/pharmaceutics16060702/s1, Table S1: Drug concentration values for patients receiving the adalimumab biosimilar; Table S2: Drug concentration values for patients receiving the etanercept biosimilar; Figure S1: Observed values of the adalimumab biosimilar concentrations versus population model-predicted values (PRED) and individual prediction values (IPRED); Figure S2: Distribution of population (PWRES) and individual weighted residuals (IWRES) versus individual predictions and normalised prediction distribution error (NPDE) for the adalimumab biosimilar concentrations; Figure S3: Visual predictive check (VPC) of popPK model fit for the adalimumab biosimilar; Figure S4: Observed values of the etanercept biosimilar concentrations versus population model-predicted values (PRED) and individual predicted values (IPRED); Figure S5: Distribution of PWRES and IWRES versus individual predictions and NPDE for the etanercept biosimilar concentrations; Figure S6: VPC of popPK model fit for the etanercept biosimilar.
