# Supplementary material for: Population Pharmacokinetic Analysis and Simulation of Alternative Dosing Regimens for Biosimilars to Adalimumab and Etanercept in Patients with Rheumatoid Arthritis
Source: Pharmaceutics. 2024 May 23;16(6):702. doi: 10.3390/pharmaceutics16060702 (PMC11206727; doi:10.3390/pharmaceutics16060702)
Supplement: Supplementary file 1 [file pharmaceutics-16-00702-s001.zip › pharmaceutics-2992964-supplementary.pdf]

SUPPLEMENTARY METHODS

Sampling schedule determination

Optimal sampling time intervals were proposed using previously defined popPK models from the literature in PopDes software, an application software that can be utilised for determining optimal sampling times or windows for popPK studies (Gueorguieva *et al*. A program for individual and population optimal design for univariate and multivariate response pharmacokinetic-pharmacodynamic models. *Comput Methods Programs Biomed* 86(1), 51-61 (2007). PopDes was developed and programmed in MATLAB v.6.1. Programming utilised object-oriented features, meaning that the program can be compiled and run as a standalone Windows program with a graphical user interface. The program source code and compiled version are available upon request to Dr Kayode Ogungbenro.

The program contains an in-built library of pharmacokinetic (PK) models, and can also accommodate user-specified models. It includes three algorithms for optimisation, namely, simplex, hybrid (simplex and simulated annealing) and exchange. It is also possible to implement Bayesian criteria in a multi-response model to account for uncertainty regarding initial parameter estimates. The software was validated using Lilly TGF-β data, and its development and functions are outlined in more detail in the above reference.

Measurement of drug levels

Drug level measurements were carried out using commercially-available ELISA-based test kits produced by Grifols International, SA (Barcelona, Spain). The Promonitor®-ADL-1DV kit was used to measure Amgevita (adalimumab biosimilar) drug levels, and the Promonitor®-ETN-1DV kits was used to measure Benepali (etanercept biosimilar) drug levels. Standard laboratory equipment and a spectrophotometer (SpectraMax® Plus 384 Microplate Reader, Molecular Devices, LLC, San Jose, California, USA) were used during the experimental procedure. Samples were defrosted for two hours at room temperature, prior to thorough mixing before the experimental procedure.

Serum drug levels were measured using 96-microwell ELISA plates, which were pre-coated with anti-adalimumab and anti-etanercept human monoclonal antibody, according to which drug was being measured. Patient samples were diluted to 1:50 concentration using a dilution buffer and were transferred to separate wells. Pre-diluted calibration samples and positive and negative controls were also included for purposes of quantification of results and quality control; these were also transferred to separate wells. Any drug present in the patient samples, calibration samples and controls became bound to the immobilised anti-drug antibodies during an incubation period of one hour at room temperature. Following incubation, any unbound material was removed by washing the wells with a 20X buffer containing phosphate-buffered saline and tween-20. Each well was then loaded with a second horseradish peroxidase-labelled anti-drug monoclonal antibody to form a sandwich complex. The plate was incubated for a further one hour at room temperature to allow the labelled antibody to bind to the drug attached to the microwells. Unbound enzyme-labelled antibody was again washed away with wash buffer, and a substrate of pre-diluted stabilised tetramethylbenzidine was added to measure enzyme activity. After 15 minutes, a stop reagent of pre-diluted sulphuric acid solution was added to halt the reaction. Colour intensity as a result of the enzymatic reaction was measured in triplicate using a spectrophotometer at wavelength 450nm. The generated optical density values were proportional to the drug concentration in each sample.

Softmax Pro 7 software (compatible with the SpectraMax® Plus 384 Microplate Reader, Molecular Devices, LLC, San Jose, California, USA) was used to interpolate the optical density values and determine drug level concentrations. Interpolated values were multiplied by the dilution factor (x50) to obtain drug levels in patient samples.

PopPK analysis

For each drug studied, one-, two- or three-compartment mammillary models assuming first-order absorption and elimination were tested. Estimated PK parameters were given as apparent values, due to extravascular administration via the subcutaneous route. PK parameters were parameterised as clearance (CL) and volume of distribution (V_D). Structural models were compared using the Akaike information criterion (AIC). BSV in PK parameters was described using an exponential model. For parameters where BSV could not be estimated, this was removed from the analysis and therefore, only typical individual values were estimated. Correlations between parameters were investigated. Additive, proportional or combined additive and proportional models were tested for residual unexplained variability (RUV). Three covariates were tested: age and body weight (continuous covariates) and sex (binary covariate). Covariate models were compared using both –2 log-likelihood (–2LL) and AIC. Models with the lowest significant –2LL value (assessed using a likelihood ratio χ^2 test, LRT) and the lowest AIC, with the simplest combination of covariates and between-variable correlations, were selected.

Goodness-of-fit (GOF) was visually assessed using plots of:

- Population-predicted (PRED) and individual-predicted (IPRED) measurements versus observed measurements (DV).
- IPRED and DV versus time.
- Residuals, represented in plots of:
    - Population weighted residual distributions (PWRES).
    - Individual weighted residual distributions (IWRES).
    - Normalised prediction distribution errors (NPDE). Distribution was tested using the Shapiro-Wilk test at a level of $\alpha = 0.05$.

SUPPLEMENTARY RESULTS

Table S1. Drug concentration values for patients receiving the adalimumab biosimilar.

ID	Time (hours)	Drug Concentration (µg/mL)	Concentration Inside/Above Therapeutic Window? (5 – 8 mg/L)
1	0.00	0.135	No
1	1.00	0.161	No
1	335.58	3.147	No
1	671.83	6.290	Yes
1	1007.75	6.499	Yes
1	2007.60	10.156	Yes
2	0.00	0.124	No
2	0.82	0.115	No
2	335.92	0.853	No
2	672.33	0.961	No
2	1008.17	1.173	No
2	2016.02	0.369	No
3	0.00	0.137	No
3	1.08	0.157	No
3	334.53	2.464	No
3	670.50	6.689	Yes
3	1006.48	9.014	Yes
3	2182.55	7.690	Yes
4	0.00	0.120	No
4	1.08	0.124	No
4	338.13	1.084	No
4	674.15	3.853	No
4	1010.25	5.842	Yes
4	202.20	7.806	Yes
5	0.00	0.116	No
5	1.05	0.123	No
5	333.55	3.126	No
5	669.95	4.710	No
5	1005.23	6.908	Yes
5	2013.65	11.860	Yes
6	0.00	0.114	No
6	1.03	0.178	No
6	1005.38	2.504	No
6	2013.50	4.422	No
7	0.00	0.117	No
7	0.97	0.121	No
7	332.48	4.295	No
7	668.63	7.522	Yes
7	1004.63	6.849	Yes
7	2012.77	14.477	Yes
8	0.00	0.112	No
8	1.00	0.155	No
8	327.27	4.428	No
8	663.27	7.654	Yes
8	999.48	7.807	Yes
8	2007.38	9.199	Yes

9	0.00	0.119	No
9	1.00	0.160	No
9	358.77	3.304	No
9	669.57	2.784	No
9	1034.43	3.658	No
9	2686.47	4.777	No
10	0.00	0.115	No
10	1.57	0.123	No
10	332.72	4.254	No
10	692.63	7.616	Yes
10	1004.62	9.161	Yes
10	2012.72	10.653	Yes

Therapeutic window as per: Pouw MF, Krieckaert CL, Nurmohamed MT *et al*. Key findings towards optimising adalimumab treatment: the concentration-effect curve. *Ann Rheum Dis* 74(3), 513-518 (2015).

Table S2. Drug concentration values for patients receiving the etanercept biosimilar.

ID	Time (hours)	Drug Concentration (μg/mL)	Concentration Inside/Above Therapeutic Window? (2.1 – 4.7 MG/L)
1	0.00	0.017	No
1	116.32	7.571	Yes
1	332.72	8.504	Yes
1	669.87	8.701	Yes
1	1005.77	5.595	Yes
1	2013.87	8.784	Yes
2	0.00	0.016	No
2	1.00	0.027	No
2	142.98	2.968	Yes
2	334.43	2.819	Yes
2	838.52	4.384	Yes
2	1006.32	4.119	Yes
2	2014.28	3.521	Yes
3	0.00	0.014	No
3	1.02	0.028	No
3	140.55	5.538	Yes
3	329.13	9.163	Yes
3	663.63	9.453	Yes
3	999.70	10.307	Yes
3	2007.70	9.170	Yes
4	0.00	0.013	No
4	1.00	0.041	No
4	143.17	3.902	Yes
4	335.42	8.234	Yes
4	671.35	4.307	Yes
4	1007.35	5.751	Yes
4	2015.38	1.757	No
5	0.00	0.021	No
5	1.00	0.049	No
5	164.98	3.770	Yes
5	332.88	3.800	Yes
5	668.57	5.463	Yes
5	1004.43	4.677	Yes
5	2012.48	5.216	Yes
6	0.00	0.017	No
6	167.75	4.692	Yes
6	335.77	4.454	Yes
6	671.80	0.015	No
6	1007.92	4.189	Yes
6	2687.87	4.876	Yes

Therapeutic window as per: Jamnitski A, Krieckaert CL, Nurmohamed MT *et al*. Patients non-responding to etanercept obtain lower etanercept concentrations compared with responding patients. *Ann Rheum Dis* 71(1), 88-91 (2012).

Figure S1. Observed values of the adalimumab biosimilar concentrations versus population model-predicted values (PRED) and individual prediction values (IPRED).

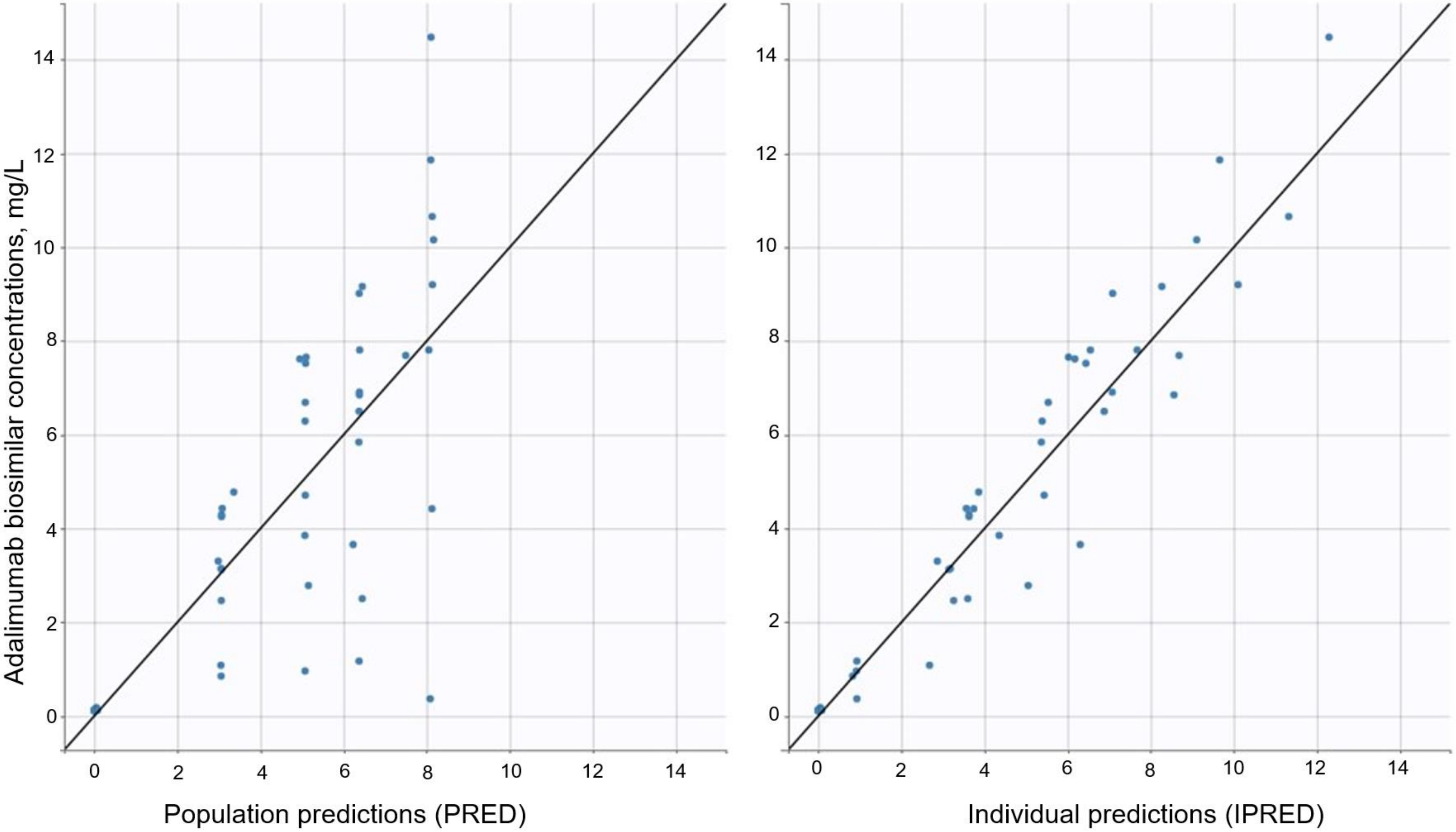

Figure S2. Distribution of population (PWRES) and individual weighted residuals (IWRES) versus individual predictions and normalised prediction distribution error (NPDE) for the adalimumab biosimilar concentrations.

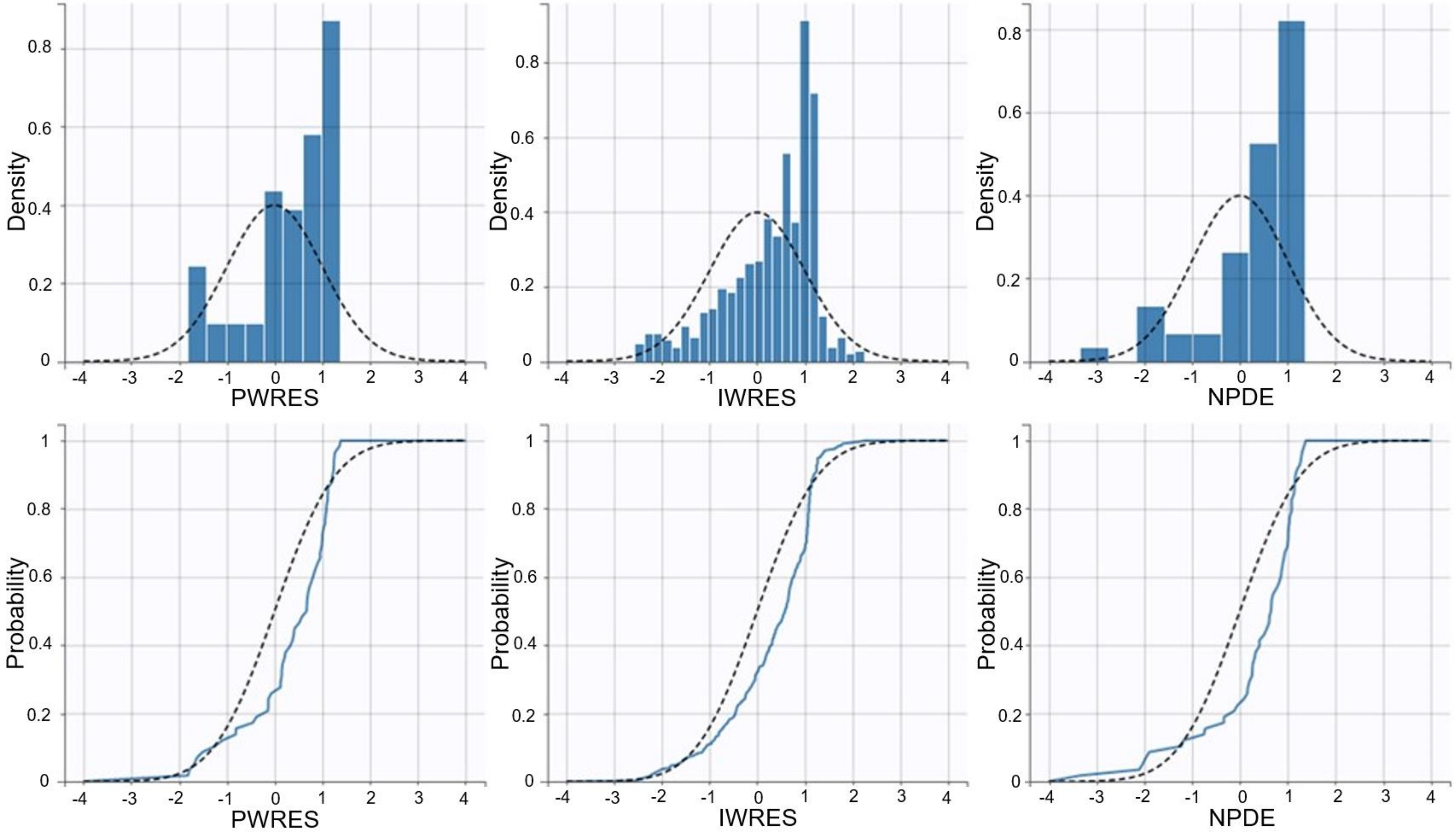

Figure S3. Visual predictive check (VPC) of popPK model fit for the adalimumab biosimilar.

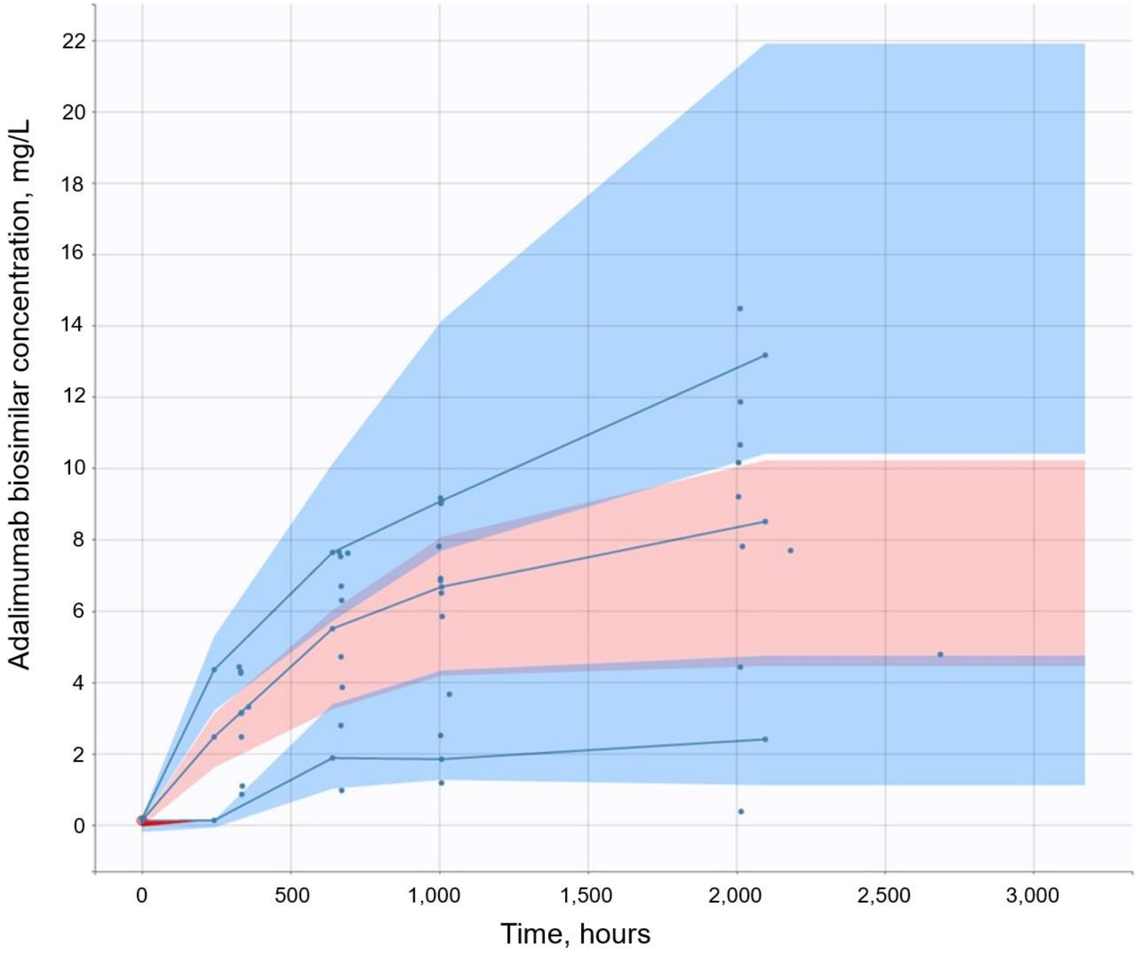

INTERPRETATION OF VISUAL PREDICTIVE CHECK: The light red zone represents a simulation-based 95% confidence interval (CI) around the median adalimumab biosimilar concentration for the population, which is denoted by the middle solid blue line. The 10th and 90th percentiles are represented by the lower and upper solid blue lines, respectively, and their 95% CI are represented by the surrounding light blue areas. Outliers are highlighted with the bright red area. Solid blue circles are the actual adalimumab biosimilar concentration values of the sample population.

Figure S4. Observed values of the etanercept biosimilar concentrations versus population model-predicted values (PRED) and individual predicted values (IPRED).

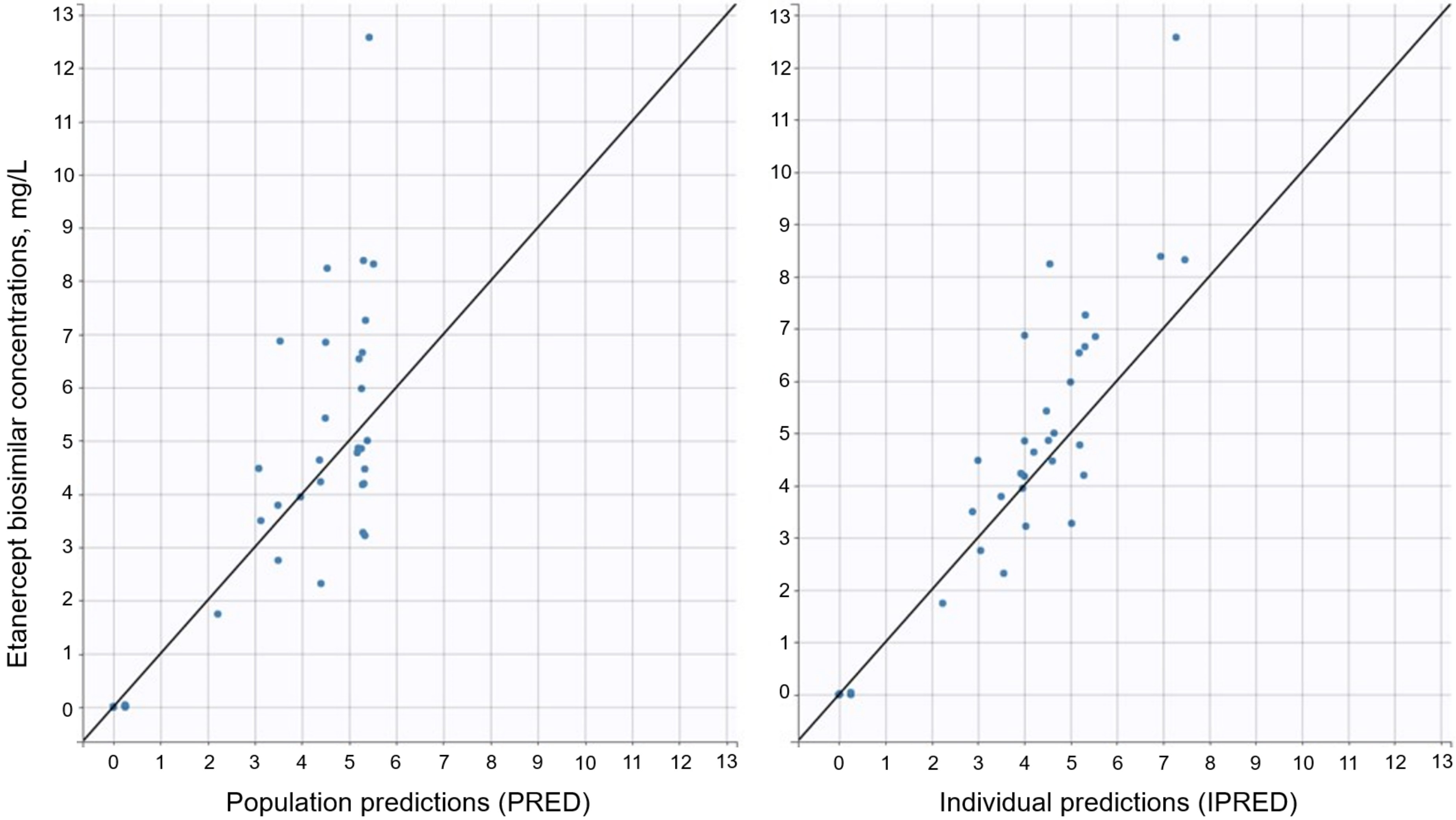

Figure S5. Distribution of PWRES and IWRES versus individual predictions and NPDE for the etanercept biosimilar concentrations.

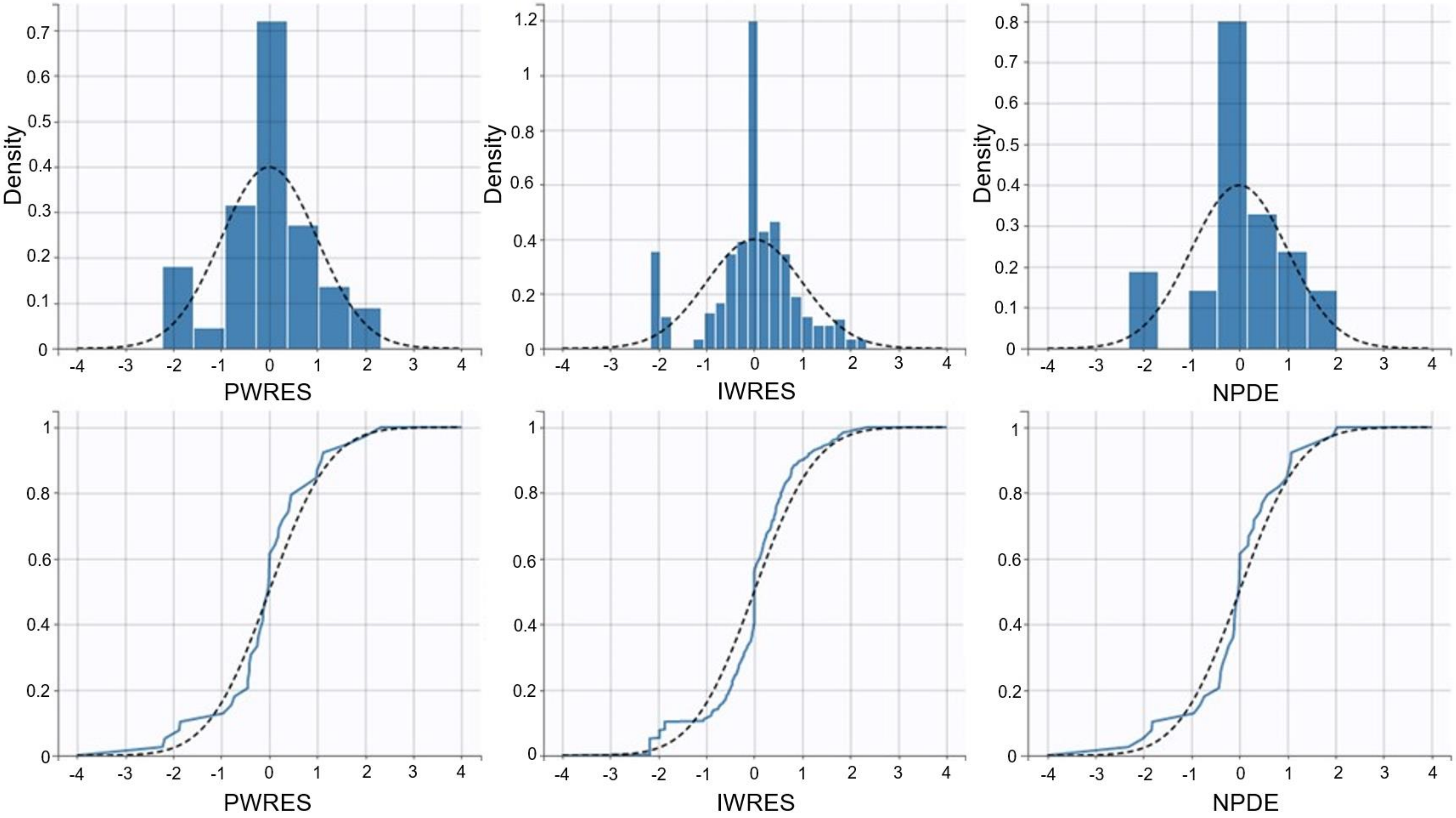

Figure S6. VPC of popPK model fit for the etanercept biosimilar.

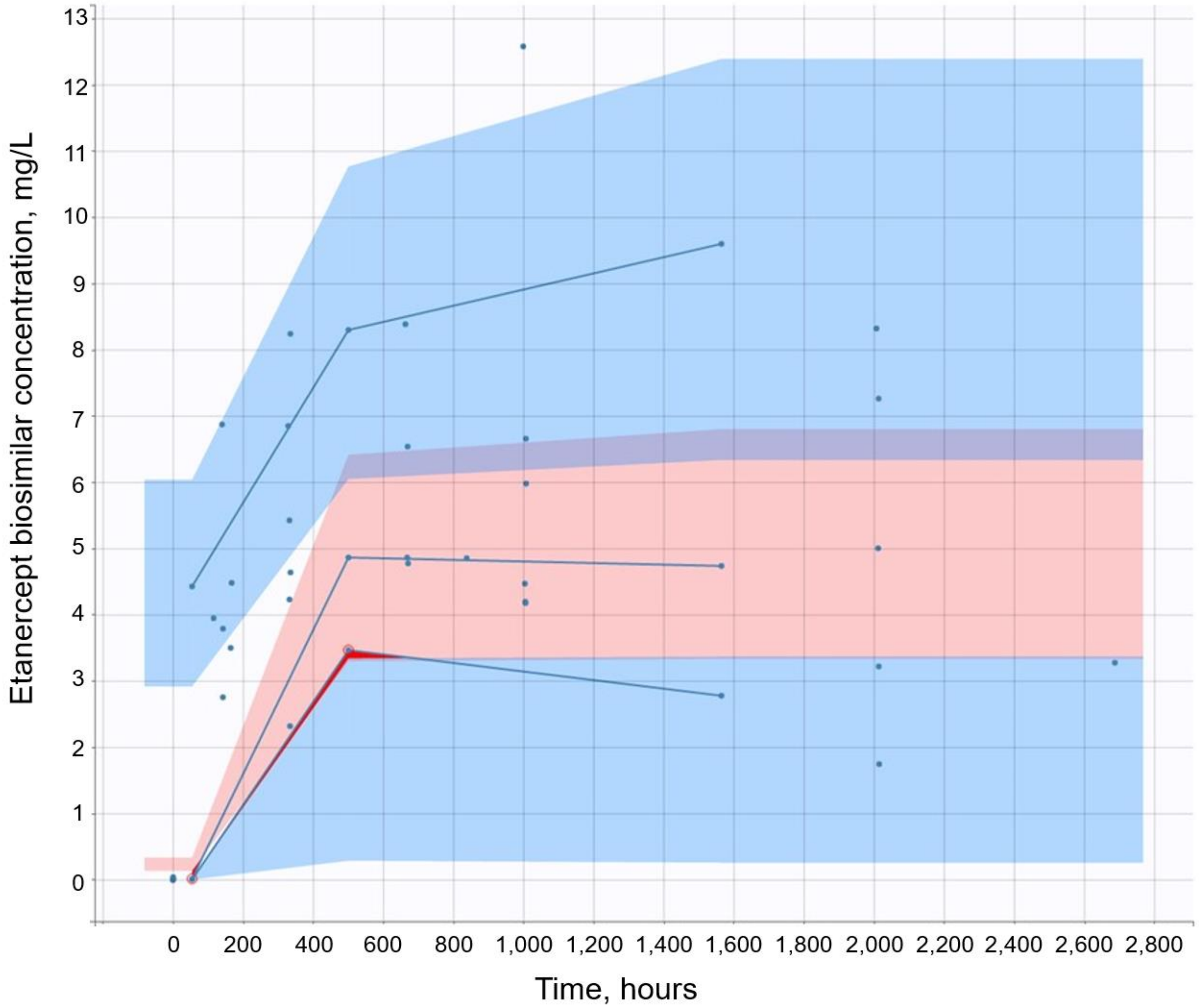

INTERPRETATION OF VISUAL PREDICTIVE CHECK: The light red zone represents a simulation-based 95% CI around the median etanercept biosimilar concentration for the population, which is denoted by the middle solid blue line. The 10th and 90th percentiles are represented by the lower and upper solid blue lines, respectively, and their 95% CI are represented by the surrounding light blue areas. Outliers are highlighted with the bright red areas. Solid blue circles are the actual etanercept biosimilar concentration values of the sample population.
